# Identification and correction of systematic error in high-throughput sequence data

**DOI:** 10.1186/1471-2105-12-451

**Published:** 2011-11-21

**Authors:** Frazer Meacham, Dario Boffelli, Joseph Dhahbi, David IK Martin, Meromit Singer, Lior Pachter

**Affiliations:** 1 Department of Mathematics, University of California, Berkeley, 970 Evans Hall #3840, Berkeley, CA 94720 USA; 2 Children's Hospital Oakland Research Institute, 5700 Martin Luther King Jr Way, Oakland, CA 94609 USA; 3 Computer Science Division, University of California, Berkeley, 387 Soda Hall, Berkeley, CA 94720 USA; 4 Department of Molecular & Cell Biology, University of California, Berkeley, 142 LSA #3200, Berkeley, CA 94720

## Abstract

**Background:**

A feature common to all DNA sequencing technologies is the presence of base-call errors in the sequenced reads. The implications of such errors are application specific, ranging from minor informatics nuisances to major problems affecting biological inferences. Recently developed "next-gen" sequencing technologies have greatly reduced the cost of sequencing, but have been shown to be more error prone than previous technologies. Both position specific (depending on the location in the read) and sequence specific (depending on the sequence in the read) errors have been identified in Illumina and Life Technology sequencing platforms. We describe a new type of *systematic *error that manifests as statistically unlikely accumulations of errors at specific genome (or transcriptome) locations.

**Results:**

We characterize and describe systematic errors using overlapping paired reads from high-coverage data. We show that such errors occur in approximately 1 in 1000 base pairs, and that they are highly replicable across experiments. We identify motifs that are frequent at systematic error sites, and describe a classifier that distinguishes heterozygous sites from systematic error. Our classifier is designed to accommodate data from experiments in which the allele frequencies at heterozygous sites are not necessarily 0.5 (such as in the case of RNA-Seq), and can be used with single-end datasets.

**Conclusions:**

Systematic errors can easily be mistaken for heterozygous sites in individuals, or for SNPs in population analyses. Systematic errors are particularly problematic in low coverage experiments, or in estimates of allele-specific expression from RNA-Seq data. Our characterization of systematic error has allowed us to develop a program, called SysCall, for identifying and correcting such errors. We conclude that correction of systematic errors is important to consider in the design and interpretation of high-throughput sequencing experiments.

## Background

The technological advances that have produced "the third phase of human genomics": sequencing of individual genomes and the determination of rare variants across populations by enabling whole genome sequencing at low cost [1], are accompanied by higher error rates [2,3]. Improved statistical methods that accommodate these high error rates are needed in the calling of heterozygous sites from low coverage data [1]. The design of effective statistical methods requires precise characterization of error in high-throughput sequence data. Previous work has examined the behavior of individual base-call errors in sequence reads [3-5]. In this paper we discuss a previously undescribed phenomenon in sequence data where these base-call errors aggregate at specific genomic locations across multiple sequence reads. We focus on Illumina technology, although we have observed systematic error on other platforms and return to this in the Discussion.

**Figure 1:**
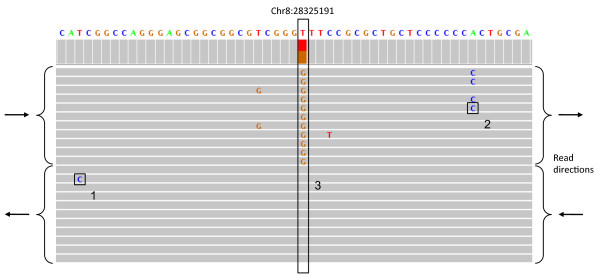
**Types of errors**. A screenshot from the IGV browser [21] showing three types of error in reads from an Illumina sequencing experiment: (1) A random error likely due to the fact that the *position *is close to the end of the read. (2) Random error likely due to *sequence specific *error- in this case a sequence of Cs are probably inducing errors at the end of the low complexity repeat. (3) *Systematic error*: although it is likely that the GGT sequence motif and the GGC motifs before it created phasing problems leading to the errors, the extent of error is not explained by a random error model. In this case, all the base calls in one direction are wrong as revealed by the 11 overlapping mate-pairs. In particular, all differences from the reference genome are base-call errors, verified by the mate-pair reads, which do not differ from the reference. Given the background error rate, the probability of observing 11 *error-pairs *at a single location, given that 11 mate-pair reads overlap the location, is 1.5 × 10^-26^. Moreover, given the presence of such errors at a single location, the probability that all of the errors occur on the same strand (i.e., on the forward mate pair) is $\frac{1}{1024}=0.00098$. Note that the IGV browser made an incorrect SNP call at the systematic error site (colored bar in top panel).

We begin by describing the types of sequencing error that have been previously characterized, and their relationship to the systematic error we have discovered. The likelihood of a base-call error occurring at any particular location in a sequence read is influenced by several parameters. It is known that base-call errors are more likely towards the ends of reads and that surrounding sequence motifs influence error frequencies [3-5]. For example, errors are more likely at positions preceded by GG or following a number of GGC motifs [5], but regardless of the preceding motif, errors become more likely towards the end of reads [3]. However, we have found that errors at some *genomic *positions appear with greater frequency than can be explained by these effects, and we refer to this as *systematic error*. Systematic error manifests as many individual base-call errors from separate sequence reads occurring at the same genomic position (Figure 1). Thus, a systematic error comprises many individual base-call errors (from different reads) that fall at the same genomic location.

These errors have the potential to be especially troublesome because they can confound methods that identify errors based on their sparsity among reads. For example, we show systematic errors affect current SNP (Single-Nucleotide Polymorphism) calling methods, where the first step involves computing the posterior probability for a SNP at every site based on relative nucleotide counts [6]. Although filters based on the depth of reads are frequently applied (mostly to screen for indels, copy number variants, or other structural variation) [7,8], most existing approaches will not identify systematic errors, or distinguish them from true SNPs. Similarly, the detection of RNA editing sites in RNA-Seq data is complicated by systematic error, because an accumulation of errors at a transcriptome site can appear to be an edit event when compared with a reference genome that may have been sequenced using another technology [9].

In this paper we present a thorough characterization of systematic errors using Illumina short-read sequencing data that is optimized for the detection of errors because of high coverage and high numbers of paired-end reads in which the paired reads overlapped. We show that systematic errors must be accounted for when annotating heterozygous alleles, and that although improved base calling software can correct a small number of systematic errors, it is not sufficient by itself. We present an efficient statistical algorithm for the detection of systematic error and use it to show that systematic errors are present in other datasets, including an RNA-Seq dataset, a viral reference genome and new Illumina HiSeq 2000 data from the 1000 genomes project.

## Results and Discussion

To investigate the types of errors present in whole-genome Illumina high throughput sequencing data, we conducted a paired-end methyl-Seq experiment on a male human individual with read length of 76 bp (Methods). A methyl-Seq experiment is ideal for investigating systematic error because the experiment results in high average coverage due to the fact that only sites cut by the restriction enzyme are assayed. The reads were mapped with Bowtie [10] allowing up to two mismatches. Our experiment spanned 29,827,077 genomic locations at an average coverage of 35.4. Due to the small fragment size in methyl-Seq experiments many of the mate-pair reads overlapped, providing for each such location two base calls sequenced from the same DNA molecule (Figure 1) albeit from different directions. We made use of this to distinguish between base-call errors and true heterozygosity calls in the following manner: each pair of bases originating from a single mate-pair and sequencing the same position was denoted a *reference-pair *if both calls agreed with the reference genome, a *SNP-pair *if both calls disagreed with the reference genome and agreed among themselves, and an *error-pair *if one of the calls agreed with the reference genome but the other did not. A *SNP-pair *could consist of two base-call errors, in the case that both of the paired reads had an error at the same location, but the probability of such an event was low and we ignored such cases in this study.

Because we focused on overlapping mate-pairs, we report all results in terms of pairs. For example, when stating coverage we state the number of pairs overlapping a site (the coverage of the systematic error location in Figure 1 is 11), and when we state a location has 40% errors it means that of the pairs overlapping the location 40% were *error-pairs*. In our experiment 3,985,926 genomic locations were covered by both reads of some mate-pair but we restricted our analysis to the 2,226,445 of these locations with a coverage depth of at least 10. These 2,226,445 genomic locations where covered by a total of 85,782,923 base-call pairs, 223,957 of which were error-pairs.

### Extent of systematic error

**Figure 2:**
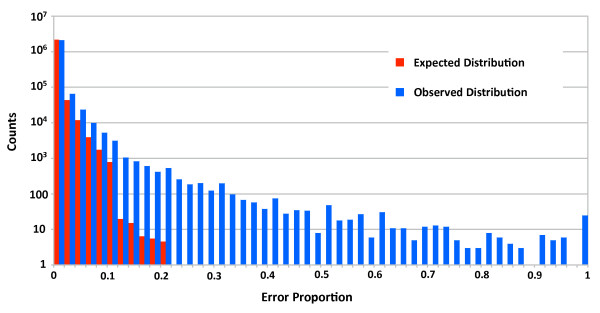
**Proportion of base call errors across genomic sites**. The observed (blue) number of locations with high base-call error frequencies significantly exceeds the expected amount (red).

We found many locations at which there seemed to be an accumulation of errors. To test the extent of this phenomenon we computed the expected number of locations with each possible proportion of error. Let *c*_10_, ..., *c*_*j*_, ..., *c*_565 _be the number of locations with coverage *j *in our data ($\sum c_{j}=2,226,445$), and $p:=\frac{\#error-pairs}{\#pairs}=0.002611$ be the probability of sequencing error. Let *B*_*i *_be a random variable for the number of locations from *c*_10_, ..., *c*_*j*_, ..., *c*_565 _with proportion of errors *i*, and let *B*_*ij *_be a random variable for the number of locations with coverage *j *and proportion of error *i*. We computed the expected number of locations to have each proportion of errors *i *as

$$E\left[B_{i}\right]=\underset{j}{\sum }E\left[B_{ij}\right]=\underset{j}{\sum }c_{j}\left(\begin{array}{c} j\\ k_{ij} \end{array}\right)p^{k_{ij}}{\left(1-p\right)}^{\left(j-k_{ij}\right)},$$

where *k*_*ij *_is the number of errors for coverage *j *that results in proportion of error *i*. Figure 2 shows a log-scale histogram of the expected and observed counts for these different error-proportions. The observed counts in the higher frequencies of errors are larger than the expected counts, indicating that there are more locations than expected that have a high frequency of base-call errors. We called such locations systematic errors, and set out to determine the characteristics of these locations, with the goal of lowering the false-positive rates in calling heterozygous sites.

For further characterization, we annotated a set of locations in which the number of *error-pairs *was significantly higher than expected, given the observed frequency of error. Setting *p = *0.002611 as in the previous section, we compute a *p-*value for a given location with *i *errors and *n *coverage as $p(K\geq i|n)=\sum_{k=i}^{n}{(}_{k}^{n})p^{k}{\left(1-p\right)}^{\left(n-k\right)}$, where *K *is a random variable indicating the number of errors at a location. Of the 2,226,445 locations with coverage of at least 10, 2,116 locations were annotated as systematic errors, using a Bonferroni correction for a 0.05 significance level. We used a Bonferroni correction because it ensures that the probability of even one false-positive is ≤ 0.05, resulting in a set that is low in false-positives, and therefore suitable for characterizing the nature of systematic error. We note that this calculation yielded a lower bound on the frequency of systematic errors in our dataset of approximately 1 in 1000 bp.

### Characterizing systematic errors

Having annotated the set of 2,116 systematic errors, we looked for characteristic features that could be identified in any high throughput sequencing experiment. Of the 2,116 sites we have determined as systematic errors, 953 had all base-call errors on the forward read and 1,062 had all base-call errors on the reverse read (an example is seen in Figure 1). We conclude from this that in systematic errors the base-call errors tend to appear on just one of the sequencing directions (forward or reverse). This tendency was noticed in [7], where the directionality on which errors occurred was used to filter false-positives from the set of heterozygous sites annotated. A possible explanation for this phenomenon is that the sequencing of some motifs, which are different on the opposite strands, have higher probability than others for base-call errors, resulting in systematic errors. This is consistent with the known overlap in absorption spectra of the G and T channels identified by a single laser in Illumina sequencing.

**Figure 3:**
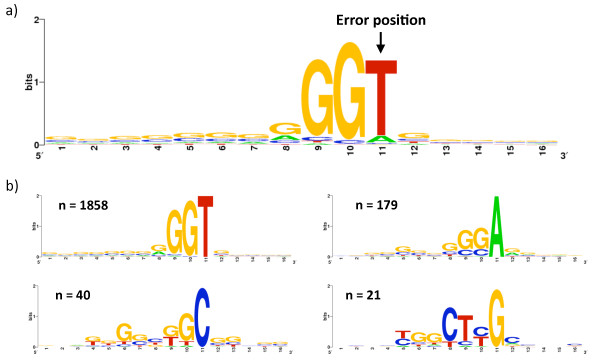
**Sequence motifs at systematic error sites**. (a) The motif around systematic errors reveals a strong enrichment for instances preceded by an occurrence of *GG *and for the error to occur at locations where the reference genome is *T*. (b) Categorized by the nucleotide at the error location. The number of systematic errors in each subset is denoted by *n*.

We therefore tested whether there are significant motifs surrounding systematic errors by generating a sequence logo [11,12] for the reference sequences around the systematic errors (Figure 3). Interestingly, we found that the first base upstream of the systematic error has greater information regarding the presence of a systematic error than the base at which the error is present. We found that the large majority of systematic errors are preceded by a *G*, and that two *G *bases followed by a *T *at the error site is by far the most common and characteristic sequence at systematic error locations. Although the *GGT *motif is a strong characteristic of systematic errors, an analysis restricted to *GGT *sites (estimating the expected error rate by that observed at *GGT*s, see Methods) showed that 660 sites, out of all 61,779 *GGT *sites, have a significant accumulation of errors. This shows that systematic errors are not accounted for by this motif alone.

**Figure 4:**
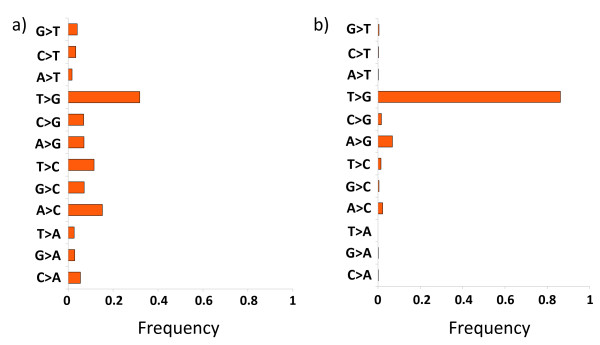
**Base substitutions of systematic errors**. Frequency of different base substitutions in (a) all errors (b) systematic errors.

To gain insight into the types of sequencing errors present at systematic errors we computed the frequencies of the different base substitutions in both systematic errors and throughout the entire dataset (Figure 4). We witnessed an extremely strong tendency for the *T *>*G *error compared to all others. Our results show that there is a higher substitution rate to *G*s than to the other nucleotides and that the substitution rate to *A *or *T *is considerably lower than the substitution rate to *C*. With respect to the reference bases at which systematic errors occur, there is a stronger tendency of error at *A *or *T *than at *C *or *G*. We divided the systematic error locations based on the reference base at which the error occurred, and tested for motifs in each of the four sets (Figure 3.b). We concluded that the strongest motif at systematic errors is that of *GGT *where the error is at the *T*, resulting in an incorrect base call of *G*.

To test whether the quality scores at the locations of systematic errors account for the extent of base-call errors observed, we computed a *p*-value for each location given its specific quality scores: Given *n *(ordered) quality scores let *K*_*i *_be a random variable for the number of errors at locations 1 to *i*, and let *X*_*i *_be an indicator variable for whether there was an error or not at location *i*. We then have that

$$p\left(K_{n}=k\right)=P\left(X_{n}=1\right)P\left(K_{n-1}=k-1\right)+P\left(X_{n}=0\right)P\left(K_{n-1}=k\right),$$

and can use dynamic programming to compute the *p*-value for each location in *O*(*n*^2^) time. Of the 2,226,445 positions with read count of at least 10, 268 had a significant accumulation of error under a Bonferroni correction for a significance level of 0.05 (the probability of even one false-positive is less than 0.05). It is interesting that significant positions were found, given that in general throughout the experiment the quality scores tend to predict a higher error rate than that observed ($\frac{\#error-pairs}{\#pairs}=0.002611$ while the quality scores predict an error-pair frequency of 0.00416).

The characteristics of systematic errors, occurring mostly at *GGT *motifs where the error that occurs is a *T *>*G *substitution, implies that the errors could be a result of the sequencing technology, which makes it hard to distinguish between a *GGG *and a *GGT *instance. It is the base-calling algorithm that makes such distinctions, given the images output from the Illumina machine. We asked whether systematic errors could be accounted for by base-callers that utilize sophisticated statistical techniques to reduce error. To test this we compared the systematic errors present in a dataset base-called by Bustard (Illumina's base-caller) to those present in the same dataset when base-called by naiveBayesCall [13], to our knowledge the most accurate base-calling algorithm available. We used for this the dataset that was used in [13] from the phiX174 virus (Methods). We found 59 systematic errors in the Bustard called dataset and 40 systematic errors in the naiveBayesCall dataset, amounting to a systematic error rate of 1 in 91 bp and 1 in 135 bp respectively. We believe the higher frequency of systematic errors is due to the phiX174 genome being richer than human in *GGT *motifs (data not shown) and to the high sequencing coverage (see Conclusions section). These results show that while systematic error can be reduced with more sophisticated base calling, it is a persistent problem at a significant level even when using state of the art methods.

To test replicability of the locations at which systematic errors occur, we conducted a second methyl-Seq experiment on the same individual (Methods). The error frequency in this second experiment was determined as $p=\frac{\#error-pairs}{\#pairs}=0.00162$ and of the 2,419,666 locations with coverage of at least 10 pair-calls, 3,272 locations were annotated as systematic errors using a Bonferroni correction of 0.05. From the 2,160,736 positions with at least 10 pair-calls in both of the experiments, 1,916 and 2,519 were annotated as systematic errors in the first and second experiments, respectively, and of those 1,279 locations were annotated as systematic errors in both experiments. This shows that while there is some variability in the locations determined as systematic errors, locations at which systematic errors occur are highly replicable (the expected number of systematic errors to be called at the same locations is 2). We tested whether the significant overlap of the locations at which systematic errors were detected was due to *GGT *motifs being more prone for systematic errors than other motifs. Of the 61,779 *GGT *sites that were overlapped by at least 10 pair-calls in each experiment, 1,596 and 2,080 locations were annotated as systematic errors in the first and second experiments, respectively, and of these 1,095 locations were annotated as systematic errors in both experiments (the expected number of systematic errors to be called at the same locations when restricting to *GGT *positions is 54). The lists of systematic errors for both experiments are available at: http://bio.math.berkeley.edu/SysCall/systematic_error_lists/.

### Identification and correction of systematic errors

The main concern regarding systematic errors is that they may be incorrectly annotated as heterozygous sites in an individual or as rare variants in a population. Fortunately, in systematic error the extent of error at a location usually does not result in an equal ratio of reference to non-matching reference calls, making it easier for methods that expect such a ratio to identify these sites as non-SNPs. Nonetheless, SAMtools [6] identified 12 of the 2,116 systematic errors in our methyl-Seq dataset as SNPs (three of these are annotated as SNPs in dbSNP130), and in the SNP-calling procedure for the 1000 genomes project a filtering step based on directionality of sequencing was used to account for systematic errors (supplementary material of [7]). Systematic error may pose an even greater difficulty in population studies, where allele ratios are not expected to be 1:1. This difficulty also arises in RNA-Seq experiments in which variants are annotated alongside expression levels [14]. Systematic error may also affect RNA-Seq experiments in the bias it can introduce in coverage at systematic error sites. Such bias can in turn affect expression level estimates [15].

**Figure 5:**
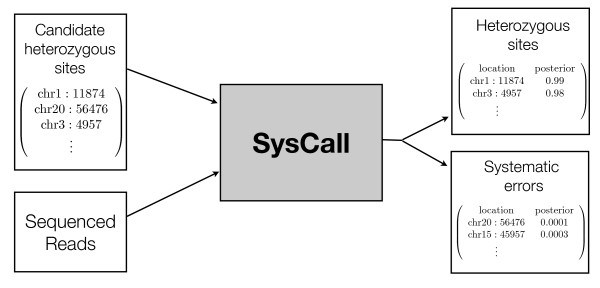
**Using SysCall to distinguish heterozygous sites from systematic errors**. SysCall takes as input a list of genomic locations indicating candidate heterozygous sites and the reads sequenced from the experiment (in SAM format), and divides the initial candidate list into two lists: a list of heterozygous sites and a list of systematic errors, printing next to each site its posterior probability of being a true heterozygous site.

To account for this we have designed SysCall - a classifier which given a list of potential heterozygous sites and the reads from an Illumina experiment classifies each location as a systematic error or a heterozygous site (Figure 5). Our classifier uses logistic regression to combine the different characteristics of systematic errors and make predictions (Methods). Importantly, SysCall does not assume that the experiment preformed is paired-end or that the expected frequency of variant observations is half, making it applicable to the different types of high throughput experiments discussed.

#### Assessing SysCall's performance

In order to test SysCall's performance we annotated a set of locations in our methyl-Seq dataset that would be candidates for heterozygous sites (where a significant amount of the base-calls differ from the reference) and for which using the overlap between paired reads we could call as systematic errors or heterozygous sites with high certainty. We used the same sets of locations that were annotated for training SysCall (Methods): a "SNPs" set consisting of 491 locations and a "Systematic errors" set consisting of 338 locations. From each mate-pair one of the reads was chosen at random to simulate a non-overlapping (and non paired-end) dataset.

As a first test of our classification algorithm we ran 100 iterations in which we generated training and test sets by randomly dividing the "SNPs" and "Systematic errors" sets into halves (from each of the "SNPs" halves 169 instances were randomly selected in order to have the same number of systematic errors and SNPs in the training and test sets). In each iteration we generated a feature matrix for the training and test sets, learned the coefficients of the logistic regression classifier from the training set, and classified the instances of the test set, recording the percentage of instances that were classified correctly (as either systematic errors or heterozygous sites). The distribution of the percentage of instances classified correctly from the 100 iterations had a mean of 99.0% and a standard deviation of 0.005.

A strong characteristic of systematic errors is that the differences from the reference have a strong bias to occur on either the forward or reverse direction. We tested the ability to classify locations using the same logistic regression classifier but using only the directionality bias feature: *u*_*l *_= (*q*_*l*1 _- *q*_*l*2_). When running 100 iterations of training and testing as before using this classifier, the distribution of the percentage of instances classified correctly had a mean of 72.1% and a standard deviation of 0.021. Therefore, a significant amount of precision is gained when making use of all six features in the classification process. This is mostly due to an increase in the recall rate of the classifier, where SNPs that are annotated as systematic errors when using only the directionality bias criterion are recognized as SNPs when making use of all features.

**Figure 6:**
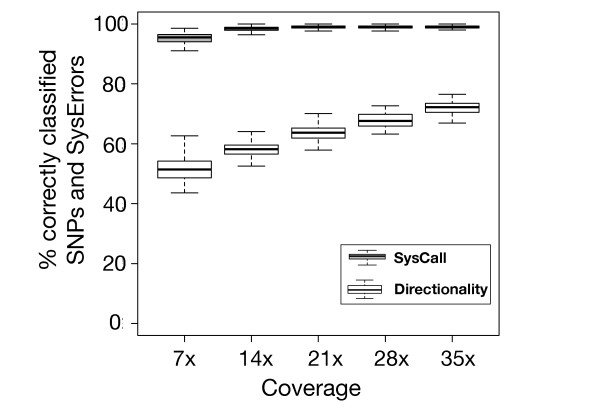
**SysCall accurately distinguishes heterozygous sites from systematic errors**. Proportion of correctly classified instances at different sequencing coverages for SysCall (grey) and for a logistic regression classifier that uses only the feature of directionality difference in error frequency (white).

A main purpose when designing SysCall was to be able to distinguish systematic errors from heterozygous sites in datasets of lower coverage than that available to us (35.4×). To evaluate SysCall's performance on different coverage depths, we simulated experiments of lower coverage by randomly sampling a given percentage from the initial set of reads. For each of 20%, 40%, 60% and 80% (resulting in coverage of 7×, 14×, 21×, and 28× respectively), we ran 100 iterations where in each iteration we randomly chose the given percentage from our reads, refined our set of locations to those with at least one base-call differing from the reference and proceed as in the previous test: divide the locations into a training and test set (the number of instances in each being half of the smaller sized set), compute features, train, classify, and record the percentage of instances classified correctly. The results for these tests, together with the results for the same tests when using only the directionality bias feature for classification are shown in Figure 6. SysCall's classifications are highly accurate at all of the coverage rates tested, and the improvement relative to using only the directionality bias is negatively correlated with the mean coverage rate, as expected.

To assess SysCall's ability to detect false-positives in SNP calls from Illumina datasets, we analyzed the GAII sequencing data available for NA18507, chromosome 21 [16]. SAMtools called 61,867 SNPs in the dataset and SysCall partitioned those locations into a set of 61,390 SNPs and 477 systematic errors. As a "gold standard" dataset we used the SNP calls for individual NA18507 available from the HapMap project [17]. From the set of SNPs called by SAMtools 11,984 (19.37%) were present in the "gold standard" dataset. Of the 61,390 SNPs called by SysCall 11,973 (19.50%) were in the "gold standard" set. Of the 477 systematic errors 11 (2.3%) were in the "gold standard" set. Our results show that SysCall helps clean the set of SNPs called by SAMtools from false-positives. We note that in this analysis half of the reads, in expectation, are expected to differ from the reference. When searching for variants in experiments where this is not the case (such as RNA-Seq, methyl-Seq, rare variant detection etc.) it is easier to mistake systematic errors for true variants and in such cases we expect SysCall's contribution will be even greater.

### Presence of systematic errors in other datasets

In order to verify that systematic errors are not specific for the methyl-Seq procedure we looked for evidence of systematic errors in other high throughput datasets. We believe systematic error will be extremely important to correct for in RNA-Seq experiments, in which one attempts to annotate both heterozygous sites and expression levels to derive allele specific expression estimates. We therefore looked for systematic errors in the RNA-Seq dataset from Ambion Human Brain Reference by Illumina (accession SRA012427), on chromosome 1. Since this dataset did not contain overlapping paired reads we could not annotate *error-pairs*. Instead, we used directionality bias of the base-calls different from the reference to annotate systematic error. We could do so because the coverage in this dataset is high (at transcripts that are highly expressed). For each of the 857,570 locations covered by at least 10 forward and 10 reverse reads we conducted a chi-square test, testing for association between occurrence of mismatches and directionality of sequencing. Under a Bonferroni correction for a 0.05 significance level, we found 991 systematic errors. Thus we have approximately 1 in 1000 sites that are shown to be systematic errors. The method used here, using directionality bias, is statistically weaker than the method with which we identified systematic errors from the methyl-Seq experiment, where we used overlapping mate-pairs to identify base-call errors. The fact that the frequency of identified systematic errors in the RNA-Seq dataset is as high as in the methyl-Seq dataset implies that there are more systematic errors present in the RNA-Seq data than in the methyl-Seq data; this could be due to this dataset being produced by an older version of Illumina's GA.

**Figure 7:**
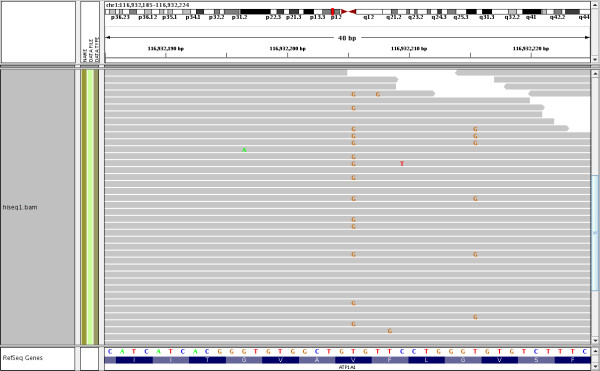
**Systematic errors in HiSeq data**. A screenshot from the IGV browser [21] showing two systematic errors in the HiSeq dataset analyzed. These locations are not statistically significant under a chi-squared test for directionality bias (after correcting for multiple hypotheses), demonstrating the weakness of this test.

We also looked at newer Illumina data generated by the HiSeq 2000 machines as part of the 1000 genomes project [7]. We analyzed exome data from chromosome 1 (accession ERX01220). We aligned reads to the reference genome with Bowtie and refined our analysis to the 848,742 sites that were covered by at least 10 reads in each direction. When conducting the same statistical test as for the RNA-Seq data, only 2 sites were determined as statistically significant with respect to the differences from the reference being present on one of the sequencing directions. However, testing for directionality bias of mismatches in this way has little power, and many strong systematic errors are missed by this method (Figure 7). This results in many locations that are not detected by this method as systematic errors but would be wrongly annotated as heterozygous sites due to their characteristics. We therefore annotated a set of candidate heterozygous sites as those locations with at least 10% of the base-calls being different from the reference sequence and with at least 5 differences from the reference, resulting in a set of 1,712 locations. Running SysCall on this set, 316 locations were classified as systematic errors. When annotating SNPs in the 1000 genomes project a filtering step was applied, detailed in sections 5.1.1 and 5.2.1 of the supplementary information of [7], designed specifically to filter out locations in which the base-calls different from the reference are not evenly distributed between the forward oriented and reverse oriented reads. The filtering step applied in [7] to avoid calling systematic errors as SNPs can decrease the number of false-positive SNP calls, but relies on having a sufficient number of reads from each strand and makes use only of the strand-specific characteristic of systematic errors. As we have shown, distinguishing between systematic errors and heterozygous sites can be greatly improved by taking additional evidence into account.

## Conclusions

We have identified systematic error in Illumina sequence that is prevalent in different types of datasets, and that does not appear to be easily correctible during base-calling. This systematic error has significant implications for SNP calling, especially at low coverage [18]. Moreover, while increasing the extent of coverage enables the detection of rare variants in population studies and low expression rates in transcriptome studies, it also reveals locations of weaker systematic errors (locations at which there is a small accumulation of base-call errors). Thus, the problem of distinguishing systematic error from true heterozygous sites persists regardless of the extent of coverage. We detected this type of error, and could thoroughly characterize it, thanks to a dataset with overlapping paired-end reads and with very high coverage. Making use of our characterization we have designed and implemented a classifier to correct for systematic errors at much lower coverage depths and with no need for paired-end reads. We have shown that by using the different characteristics in the prediction process we gain a significant increase in performance over using directionality bias alone.

Although we have provided a preliminary characterization of systematic error, with further work and additional data it may be possible to better identify sequences associated with error. In particular, it should be possible to identify and characterize systematic error resulting from other sequencing technologies. Although such a comprehensive assessment is beyond the scope of this study, we have looked at RNA-Seq SOLiD data from [19] and have identified statistically significant systematic error. At the same time, we believe that as sequencing technology improves systematic errors should decrease, and we have observed this to be the case based on the Illumina samples we have investigated. Sequence from two years ago shows higher systematic error rates than recently sequenced data. Nevertheless, we believe that systematic error is a continuing characteristic of Illumina sequence.

## Methods

### methyl-Seq experiments

The human sample was collected with IRB approval from the Children's Hospital and Research Center, Oakland. The approval was granted for a single subject to draw blood for the purpose of examining his methylome and transcriptome, with the understanding that the subject is fully aware of the implications of collecting and analyzing personal genetic data. Immediately after phlebotomy, leukocytes were isolated by Ficoll centrifugation. B cells were isolated from the leukocyte fraction with an indirect magnetic labeling system for the isolation of untouched B cells which yields highly pure B cell preparations (Miltenyi). DNA was extracted by standard methods, and digested overnight with HpaII (NEB). HpaII cuts the sequence CCGG; methylation of the central cytosine on one or both strands protects the sequence from digestion with HpaII [20]. HpaII fragments 50-300 bp in length were isolated on an agarose gel. A paired-end sequencing library was constructed with the standard Illumina kit, and sequenced on an Illumina GAIIX to collect reads of 76 bases, resulting in 15,598,990 read pairs. Read pairs that did not terminate at CCGG restriction sites were removed, leaving 14,205,350 read pairs. The reads were mapped to the human reference genome (hg18) using Bowtie [10] as single end reads allowing 3 mismatches and requiring that the alignments be unique. Those that did not align were removed and the remaining reads were mapped again, this time as paired end reads with a mismatch limit of 2. The higher mismatch limit of 3 was used in the initial alignment step to avoid having reads with more base-call errors preferentially pass the uniqueness requirement. This produced 6,939,310 aligned read pairs mapped to 313,789 distinct locations. The same procedure was followed for the second methyl-Seq experiment from monocyte DNA. The experiment generated 14,432,723 read pairs, of which 7,265,035 were ultimately mapped to 274,230 distinct locations.

### Annotating systematic errors at *GGT *sites

The error rate in our dataset at *GGT *sites was computed as $p_{GGT}:=\frac{\#error-pairs\;at\;GGT}{\#all\;pairs\;at\;GGT}=0.0194$. We tested whether there are specific *GGT *locations at which there is a significant excess of errors by computing a *p*-value for each *GGT *site, given the number of *error-pairs *and coverage at the location, using *p*_*GGT*_, and using a Bonferroni correction of 0.05. The number of significant locations remained substantial at 660, out of 61,779 *GGT *sites considered.

### Annotating systematic errors in the phiX174

To test the influence different base callers have on the extent to which systematic errors are present in a dataset we looked for systematic errors in the non-paired reads reported in [13]. In [13], several sets of base-called reads were obtained from one run of sequencing of the phiX174 genome, each using a different base calling method to process the images generated by the sequencing machine. In this work we compared two base calling methods: Bustard, which is Illumina's base-caller, and naiveBayesCall, presented in [13]. The sequencing run generated 74,686 non-paired reads, resulting in an extremely high coverage dataset for the 5,386 bp long genome.

We mapped the reads from each method to the virus genome using Bowtie, obtaining 382.2× coverage for the Bustard called reads and 394.2× coverage for the naiveBayesCall called reads. Since phiX174 is only 5,386 bp long and has been thoroughly studied for heterozygous sites due to its use as a sequencing control, we excluded the five known SNP sites from our analysis, and at the remaining sites called all base-calls that were different from the reference as base-call errors. We computed the probability of a base-call error for each dataset of mapped reads by $p=\frac{\#\;base-call\;errors}{\#\;base\;calls}$, and identified locations with a significant accumulation of errors by computing a *p*-value for every given location with *i *errors and coverage *n *as previously described in the text, using a Bonferroni correction for a 0.05 significance level. We used the frequency of base-call errors in the Bustard called reads of 0.0029 as the error probability for both datasets, since this was the higher of the two frequencies.

We found 59 systematic errors in the Bustard called dataset and 40 systematic errors in the naiveBayesCall dataset, amounting to a systematic error rate of 1 in 91 bp and 1 in 135 bp respectively. When restricting to cases in which more than 10% of the base-calls had errors we found 15 systematic errors for Bustard and 10 systematic errors for naiveBayesCall, 7 of which were at the same sites.

### SysCall's design and implementation

In this section we describe SysCall, a logistic regression classifier designed to distinguish heterozygous sites from systematic errors, based on the characteristics of systematic errors we have discussed. We will begin with describing the features used in SysCall's model, continue with how the model parameters were learned, and end with a description of the prediction procedure given a new dataset. Importantly, the special features of the methyl-Seq dataset (overlap of paired-reads and deep coverage) were used only for the first two stages. There is no need for the dataset on which SysCall is used to have such features. As we show in Figure 6, SysCall preforms well on a single-end dataset of 7x.

#### Model features

**Figure 8:**
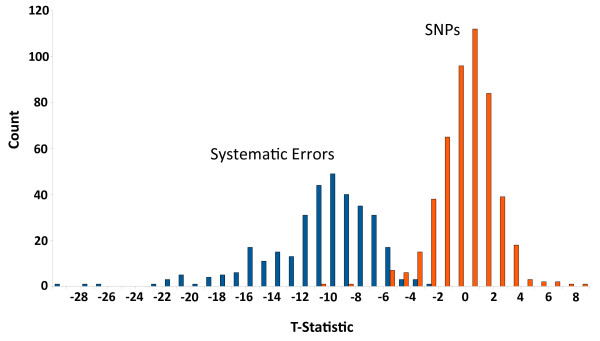
**The paired *t*-test statistic helps distinguish true SNPs from systematic errors**. The paired t-test (*PT*(*w*_0_, *w*_1_)) was computed for the "SNPs" and "Systematic errors" sets used for training SysCall. The histogram of paired t-test for the "SNPs" set (red) is centered around 0 (mean: 0.0024, std: 2.035), indicating that the quality scores at those locations were similar to their neighboring quality scores. The histogram of the "Systematic errors" set (blue) formed an almost disjoint distribution (mean: -10.505, std: 3.919).

We have chosen features to be used in SysCall based on our findings regarding the characteristics of systematic errors. Given a dataset and a location, *l*, SysCall annotates a vector of features, *x*_*l*_, as follows: First a sequencing direction is chosen (forward or reverse) as the direction with the larger proportion of base-calls that differ from the reference. SysCall only considers sites at which there is at least one base-call that differs from the reference. Let *q*_*l*1 _and *q*_*l*2 _be that proportion for the chosen and not chosen directions respectively. For example, for the location annotated as a SNP in Figure 1, we would choose the forward direction and have *q*_1 _= 1 and *q*_2 _= 0. Let *b*_*i *_be the nucleotide that is *i *places from *l *in the chosen direction and let *w*_*i *_be the vector of quality scores at the location *i *places from *l*, attained from the reads overlapping that location. A feature vector is then annotated for *l *as:

$$x_{l}=\left(b_{-2},b_{-1},b_{0},q_{l1}-q_{l2},q_{l1},\text{PT}\left(w_{0},w_{1}\right)\right),$$

where PT(*w*_0_, *w*_1_) is the paired *t*-test result on the two vectors *w*_0 _and *w*_1_. This paired *t*-test feature is computed due to our observation that the quality scores at systematic error locations tend to be lower relative to the quality scores at their neighboring sites (Figure 8), and this can help distinguish them from true heterozygous sites. As an example, for the location annotated as a SNP in Figure 1 the feature vector is (*G*, *G*, *T*, 1, 1, -5.56).

#### Parameter estimation

We learned parameters for SysCall using training sets constructed from our methyl-Seq dataset. In that dataset, due to both overlap of paired-reads and high coverage, it was possible to determine many sites with high certainty as either heterozygous sites or systematic errors. We annotated a list of locations that would be candidates for heterozygous sites (where a significant amount of the base-calls differ from the reference) and which we could call as systematic errors or heterozygous sites with high certainty. Of the 905 locations in our dataset with coverage of at least 40 (paired-calls) and at which 10-90% of the base-calls on the forward strand differed from the reference we annotated two sets: (1) "SNPs" - the 491 locations at which all differences from the reference were *SNP-pairs*. (2) "Systematic errors" - the 338 locations at which all differences from the reference were *error-pairs*. From each mate-pair one of the reads was chosen at random to simulate a non-overlapping (or non paired-end) dataset. Also, 338 locations were chosen at random for the "SNPs" set to ensure the predictions were feature-based only. A feature matrix was built for these 676 locations (the training set), and the parameters for a logistic regression model were computed by maximum likelihood estimation using R. Note that when assessing SysCall's performance the data on which the classifier was trained was different from that used to asses its performance (in each iteration only half of this dataset was used for training).

At different depths of coverage the different features may be indicative to different extents. For example, at high sequencing depths the paired *t*-test statistic and the frequency of error on each direction may have a more significant effect than at lower sequencing depths, where the sequence motif is more informative. To account for this we simulated experiments of lower coverage by randomly sampling a given percentage from the initial set of reads. For each of 20%, 40%, 60% and 80% (resulting in coverage of 7x, 14x, 21x, and 28x respectively), we randomly chose the given percentage from our reads, refined our set of locations to those with at least one base-call differing from the reference and proceeded as before to construct a different training set for every coverage.

#### Prediction procedure

SysCall takes as input a list of genomic locations and a sequencing dataset. For *n *given locations, SysCall constructs an *n *× 7 feature matrix, *M*, where *M*_*i*,* _= (1, *x*_*i*_), *x*_*i *_being the feature vector for location *i*. Then, SysCall computes the mean coverage for the given dataset and uses the model parameters learned from the training set with coverage closest to that observed, *β*, to compute the vector of posterior probabilities as

$$p_{i}=\frac{1}{1+e^{-\left[\beta^{T}M^{T}\right]i}}$$

for *i *= 1, ..., *n*. Using a threshold of 0.5 on the posterior probability, SysCall partitions the locations into "true heterozygous sites" (*p*_*i *_≥ 0.5) and "systematic errors" (*p*_*i *_< 0.5) and prints out two files accordingly, along with the posterior probability assigned to each location. In the case of multiple mappings of reads, each mapping of a read is considered by SysCall, independently of other mappings.

SysCall is implemented in R. The running time for classification is instantaneous, and the running time for feature assembly depends on the number of sequenced reads in the experiment and the number of locations considered, currently taking 10 seconds per 100,000 reads when classifying 900 locations, and is trivially parallelizable. SysCall is available at http://bio.math.berkeley.edu/SysCall/.

## Authors' contributions

FM, MS and LP formulated the problem of searching for systematic errors by studying discordant read pairs and designed a research plan. FM and MS conducted the research. DB, JD and DM performed the sequencing and contributed the datasets analyzed, and FM, MS and LP wrote the manuscript. All authors read and approved the final manuscript.

## References

[B1] Nielsen R Genomics: In search of rare human variants Nature 2010 467 7319 1050 1051 10.1038/4671050a 20981085

[B2] Hoff K The effect of sequencing errors on metagenomic gene prediction BMC Genomics 2009 10 520+ 10.1186/1471-2164-10-520 19909532 PMC2781827

[B3] Dohm JC Lottaz C Borodina T Himmelbauer H Substantial biases in ultra-short read data sets from high-throughput DNA sequencing Nucleic Acids Research 2008 36 16 e105 10.1093/nar/gkn425 18660515 PMC2532726

[B4] Taub M Bravo H Irizarry R Overcoming bias and systematic errors in next generation sequencing data Genome Medicine 2010 2 87 10.1186/gm208 21144010 PMC3025429

[B5] Nakamura K Oshima T Morimoto T Ikeda S Yoshikawa H Shiwa Y Ishikawa S Linak MC Hirai A Takahashi H Altaf-Ul-Amin M Ogasawara N Kanaya S Sequence-specific error profile of Illumina sequencers Nucleic acids research 2011 39 13 e90 10.1093/nar/gkr344 21576222 PMC3141275

[B6] Li H Handsaker B Wysoker A Fennell T Ruan J Homer N Marth G Abecasis G Durbin R 1000 Genome Project Data Processing Subgroup The Sequence Alignment/Map format and SAMtools Bioinformatics 2009 25 16 2078 2079 10.1093/bioinformatics/btp352 19505943 PMC2723002

[B7] 1000 Genomes Project Consortium A map of human genome variation from population-scale sequencing Nature 2010 467 7319 1061 1073 10.1038/nature09534 20981092 PMC3042601

[B8] Wang J Wang W Li R Li Y Tian G Goodman L Fan W Zhang J Li J Zhang J Guo Y Feng B Li H Lu Y Fang X Liang H Du Z Li D Zhao Y Hu Y Yang Z Zheng H Hellmann I Inouye M Pool J Yi X Zhao J Duan J Zhou Y Qin J The diploid genome sequence of an Asian individual Nature 2008 456 7218 60 65 10.1038/nature07484 18987735 PMC2716080

[B9] Li M Wang IX Li Y Bruzel A Richards AL Toung JM Cheung VG Widespread RNA and DNA Sequence Differences in the Human Transcriptome Science 2011 333 6038 53 58 10.1126/science.1207018 21596952 PMC3204392

[B10] Langmead B Trapnell C Pop M Salzberg S Ultrafast and memory-efficient alignment of short DNA sequences to the human genome Genome Biology 2009 10 3 R25+ 19261174 10.1186/gb-2009-10-3-r25 PMC2690996

[B11] Crooks GE Hon G Chandonia JMM Brenner SE WebLogo: a sequence logo generator Genome Research 2004 14 6 1188 1190 10.1101/gr.849004 15173120 PMC419797

[B12] Schneider TD Stephens RM Sequence logos: a new way to display consensus sequences Nucleic Acids Research 1990 18 20 6097 6100 10.1093/nar/18.20.6097 2172928 PMC332411

[B13] Kao WC Song Y naiveBayesCall: An Efficient Model-Based Base-Calling Algorithm for High-Throughput Sequencing Research in Computational Molecular Biology, Volume 6044 of Lecture Notes in Computer Science 2010 Berger B, Berlin, Heidelberg: Springer Berlin/Heidelberg 233 247

[B14] Zhang K Li JB Gao Y Egli D Xie B Deng J Li Z Lee JH Aach J Leproust EM Eggan K Church GM Digital RNA allelotyping reveals tissue-specific and allele-specific gene expression in human Nature Methods 2009 6 8 613 618 10.1038/nmeth.1357 19620972 PMC2742772

[B15] Trapnell C Williams B Pertea G Mortazavi AGK van Baren M Salzberg S Wold B Pachter L Transcript assembly and quantification by RNA-Seq reveals unannotated transcripts and isoform switching during cell differentiation Nature Biotechnology 2010 28 511 515 10.1038/nbt.1621 20436464 PMC3146043

[B16] Illumina Tru Resources Data Sets http://www.illumina.com/truseq/tru_resources/datasets.ilmn

[B17] International HapMap Project http://hapmap.ncbi.nlm.nih.gov/downloads/genotypes/latest/

[B18] Malhis N Jones S High quality SNP calling using Illumina data at shallow coverage Bioinformatics 2010 26 1029 1035 10.1093/bioinformatics/btq092 20190250

[B19] Roberts A Trapnell C Donaghey J Rinn J Pachter L Improving RNA-Seq expression estimates by correcting for fragment bias Genome Biology 2011 12 R22 10.1186/gb-2011-12-3-r22 21410973 PMC3129672

[B20] Harland RM Inheritance of DNA methylation in microinjected eggs of Xenopus laevis Proc Natl Acad Sci USA 1982 79 7 2323 2327 10.1073/pnas.79.7.2323 6285378 PMC346185

[B21] Robinson JT Thorvaldsdottir H Winckler W Guttman M Lander ES Getz G Mesirov JP Integrative genomics viewer Nat Biotech 2011 29 24 26 10.1038/nbt.1754 PMC3346182 21221095

